# Changing renal profile and early eGFR dynamics after ART initiation or switch in a large Italian HIV cohort

**DOI:** 10.3389/fmed.2026.1898349

**Published:** 2026-08-11

**Authors:** Paolo Maggi, Manuela Ceccarelli, Elena Delfina Ricci, Giancarlo Orofino, Barbara Menzaghi, Nicola Squillace, Lucia Taramasso, Giuseppe Vittorio De Socio, Stefania Piconi, Giordano Madeddu, Giovanni Francesco Pellicanò, Filippo Lagi, Antonio Di Biagio, Paolo Bonfanti

**Affiliations:** 1 Department of Medicine and Surgery, “Kore” University of Enna, Enna, Italy; 2 Fondazione ASIA, Milan, Italy; 3 Division I of Infectious and Tropical Diseases, ASL Città di Torino, Turin, Italy; 4 Unit of Infectious Diseases, ASST della Valle Olona, Busto Arsizio, VA, Italy; 5 Infectious Disease Unit, Fondazione IRCCS San Gerardo dei Tintori, Monza, Italy; 6 Infectious Diseases Unit, IRCCS Azienda Ospedaliera Metropolitana, Genoa, Italy; 7 Department of Health's Sciences, University of Genoa, Genoa, Italy; 8 Infectious Diseases Clinic, Department of Medicine and Surgery, University of Perugia, Perugia, Italy; 9 Unit of Infectious Diseases, A. Manzoni Hospital, Lecco, Italy; 10 Unit of Infectious Diseases, Department of Medicine, Surgery and Pharmacy, University of Sassari, Sassari, Italy; 11 Unit of Infectious Diseases, Department of Clinical and Experimental Medicine, AOU Policlinico G. Martino, Messina, Italy; 12 AOU Infectious and Tropical Diseases, Careggi Hospital, Florence, Italy; 13 University of Milano-Bicocca, Milan, Italy

**Keywords:** antiretroviral therapy, estimated glomerular filtration rate, HIV, pharmacovigilance, renal function

## Abstract

**Objectives:**

To assess changes in renal profile over time and short-term estimated glomerular filtration rate (eGFR) after antiretroviral treatment (ART) initiation or switch in an Italian HIV cohort.

**Methods:**

We analyzed data from SCOLTA, a multicenter prospective pharmacovigilance cohort of people with HIV (PWH) initiating or switching ART in routine care. Participants enrolled between 2007 and 2024 with baseline eGFR ≥30 mL/min/1.73 m^2^ and at least one renal assessment at 24 ± 4 weeks and/or 48 ± 4 weeks were included. Renal profile was assessed across calendar periods, and eGFR changes from baseline to week 24 and from week 24 to week 48 were evaluated using unadjusted analyses and multivariable general linear models including either drug cohort or calendar period.

**Results:**

Among 5170 participants, 25.7% were women and 18.2% were ART-naïve. Renal profile changed over time as the cohort became older and more treatment-experienced. The main decline in eGFR occurred during the first 24 weeks after ART initiation or switch, whereas changes from week 24 to week 48 were minimal. In adjusted analyses, ART-naïve participants showed a greater early decline in eGFR than ART-experienced individuals (*p* < 0.0001). TDF-associated changes were not consistent across the two adjusted models. Kidney-related treatment discontinuations were uncommon (26/5170, 0.5%).

**Conclusions:**

In PWH with baseline eGFR ≥30 mL/min/1.73 m^2^, kidney-related treatment discontinuations were uncommon. Renal profile changed over time, and the main decline in creatinine-based eGFR occurred during the first 24 weeks after ART initiation or switch, followed by relative stability between weeks 24 and 48.

## Introduction

Kidney disease remains an important comorbidity in people with HIV (PWH), contributing to morbidity and mortality even in the era of effective antiretroviral therapy (ART) (1–3). The spectrum of renal disease in PWH has changed over time. Classic HIV-associated nephropathy has become less prominent with widespread ART use, while chronic kidney disease (CKD) is now increasingly shaped by aging, hypertension, diabetes, obesity, hepatitis C coinfection, and cumulative exposure to potentially nephrotoxic antiretroviral agents (3–6). More recently, the interaction between cardiovascular, kidney, and metabolic risk in PWH has also been framed within the emerging concept of cardiovascular-kidney-metabolic syndrome, highlighting the overlap between aging, adiposity, chronic inflammation, metabolic dysfunction, and renal vulnerability (7).

Among antiretroviral drugs, tenofovir disoproxil fumarate (TDF) and some ritonavir-boosted protease inhibitors have been associated with renal safety concerns, including eGFR decline and increased CKD risk, although the magnitude and clinical relevance of these effects may vary according to baseline renal function, concomitant comorbidities, and cumulative exposure (8, 9). At the same time, interpretation of renal changes in contemporary HIV care has become more complex, because creatinine-based eGFR may be influenced not only by true changes in filtration, but also by drug-related effects on creatinine metabolism (1, 10).

The clinical context of ART use has also changed. In recent years, earlier treatment initiation, aging of the treated population, increasing multimorbidity, and the introduction of newer switch strategies have modified both the baseline renal profile of PWH and the way renal safety should be interpreted in routine practice (5, 11). Therefore, understanding whether renal presentation has changed over time, and whether early eGFR dynamics differ across ART regimens and calendar periods, remains clinically relevant.

This study investigated baseline renal profile and short-term eGFR changes in a large Italian cohort of PWH enrolled between 2007 and 2024, with follow-up assessments at 24 and 48 weeks after ART initiation or switch. The main objective was to evaluate whether creatinine-based eGFR presentation changed over time and whether short-term eGFR dynamics differed across ART regimens and calendar periods in routine clinical practice.

## Participants and methods

### Inclusion and exclusion criteria

We performed an observational analysis of prospectively collected data from the SCOLTA (Surveillance Cohort Long-Term Toxicity Antiretrovirals) project, an ongoing multicenter pharmacovigilance cohort established in 2002 to monitor adverse events (AEs) and toxicities associated with antiretroviral drugs included in the SCOLTA pharmacovigilance program in routine clinical practice (12). The project involves 25 Italian Infectious Diseases centers, and data are entered into a centralized online database (www.cisai.it).

Eligible participants were ART-naïve or ART-experienced PWH aged ≥ 18 years who entered the cohort when first prescribed one of the antiretroviral drugs included in the SCOLTA pharmacovigilance program and provided informed consent for study participation. Baseline variables included sex, age, ethnicity, weight, height, HIV-RNA, CD4 and CD8 cell counts, CDC clinical stage according to the 1993 revised classification (13), ART history, comorbidities, and concomitant non-ART therapies. Data were prospectively entered in anonymized form into a central database at 6-month intervals. Adverse events were recorded at the time they occurred.

In this analysis, we evaluated 24 and 48 week-changes in eGFR, calculated according to the 2009 CKD-EPI creatinine-based equation, and their association with ART regimen characteristics. The 2009 CKD-EPI creatinine-based equation was chosen to ensure consistency across the long study period and comparability with previous SCOLTA analyses and published HIV renal studies. Because serum creatinine was not systematically recorded before 2006, participants enrolled earlier were not eligible for the present analysis. We included all participants with a baseline eGFR ≥30 mL/min/1.73 m^2^ and at least one post-baseline renal assessment within one of the prespecified follow-up windows at 24 ± 4 weeks and/or 48 ± 4 weeks after enrollment.

### Drug cohorts and observation timepoints

Because enrollment in SCOLTA is linked to the prescription of antiretroviral drugs under surveillance, calendar period and drug cohort partially overlapped. The main drug cohorts considered in this analysis were darunavir/ritonavir (DRV/r, 2006–2013), darunavir/cobicistat (DRV/c, 2016–2018), raltegravir (RAL, 2007–2014), elvitegravir/cobicistat (EVG/c, 2012–2017), rilpivirine (RPV, 2013–2017), dolutegravir (DTG, 2014–2024), bictegravir (BIC, 2019–2024), cabotegravir (CAB, 2022–2024), and doravirine (DOR, 2020–2024).

Baseline was defined as T0, corresponding to the enrollment visit. Follow-up renal assessments were evaluated at 24 weeks ± 4 weeks (T1) and 48 weeks ± 4 weeks (T2) after enrollment. Changes in eGFR were calculated as T1 minus T0 and T2 minus T1; negative values indicated a decrease in eGFR, whereas positive values indicated an increase. When more than one creatinine value was available within a follow-up window, the value closest to the target visit was selected.

### Statistical analysis

Analyses were performed on available cases; no imputation was performed for missing renal measurements. Continuous variables were summarized as mean ± standard deviation (SD) when normally distributed and as median with interquartile range (IQR) when not normally distributed; categorical variables were summarized as counts and percentages. Distribution normality was assessed graphically using quantile-quantile plots. Baseline differences across groups were assessed using analysis of variance (ANOVA) for normally distributed continuous variables, the Kruskal-Wallis test for non-normally distributed continuous variables, and the chi-square test for categorical variables.

Within-group changes from baseline were evaluated using paired *t*-tests. Differences in eGFR changes among groups were assessed using multivariable general linear models and reported as adjusted means with 95% confidence intervals (95% CIs). Two alternative models were fitted, including either drug cohort or calendar period. In both models, covariates included ART-naïve status at baseline, BMI category (< 25 vs. ≥25 kg/m^2^; missing values were retained as a separate category), TDF use in the current regimen at baseline, and baseline eGFR. Because eGFR was calculated using the 2009 CKD-EPI creatinine-based equation, age, sex, and ethnicity were embedded in the outcome measure and were therefore not entered separately among the model covariates. Renal discontinuations were assessed over the entire available follow-up, whereas analyses of eGFR changes were limited to the prespecified windows at 24 ± 4 weeks and 48 ± 4 weeks.

All statistical tests were two-sided, and *p* values < 0.05 were considered statistically significant. Analyses were performed using SAS for Windows, version 9.4 (SAS Institute, Cary, NC, USA).

### Ethical approval

The original study protocol was approved on 18 September 2002, and four subsequent amendments were approved on 13 June 2013, 20 December 2019, 12 May 2020, and 12 June 2023 by the coordinating center, initially Hospital “L. Sacco”-University of Milan, Milan, Italy, and subsequently by all participating centers. Following the latest amendment, the coordinating center became IRCCS San Gerardo, Monza, Italy. All participants provided written informed consent, and the study was conducted in accordance with the Declaration of Helsinki and applicable Italian national regulations.

### Use of artificial intelligence tools

A large language model (ChatGPT, OpenAI) was used during manuscript preparation to assist with language editing and improvement of clarity. It was not used to generate data or perform statistical analyses. All content was critically reviewed and validated by the authors, who take full responsibility for the manuscript.

## Results

### Study population

Among 5,196 individuals with available renal data, 26 had baseline eGFR < 30 mL/min/1.73 m^2^ and were therefore excluded from the present analysis. The final study population included 5,170 participants, of whom 25.7% were women and 18.2% were ART-naïve. All 5,170 participants contributed to the baseline-to-Week 24 analysis. Of these, 4,274 participants (82.7%) also had an evaluable renal assessment at Week 48 and contributed to the Week 24-to-Week 48 analysis; Week 48 data were missing for 896 participants (17.3%).

### Baseline profile across calendar periods

Table 1 summarizes baseline characteristics across calendar periods, and Supplementary Table S1 reports subgroup-specific baseline eGFR values. Over time, the cohort became older, less frequently included people with history of intravenous drug use (IDU) and HCV-positive individuals and showed a progressively higher proportion of ART-experienced participants. Baseline eGFR was lower in participants with BMI ≥25 kg/m^2^ than in those with BMI < 25 kg/m^2^ (90 vs. 95 mL/min/1.73 m^2^, *p* < 0.0001) and in ART-experienced than in ART-naïve individuals (91 vs. 106 mL/min/1.73 m^2^, *p* < 0.0001). Participants receiving a TDF-containing regimen at enrollment had higher baseline eGFR values than those receiving other regimens (98 vs. 91 mL/min/1.73 m^2^, *p* < 0.0001).

**Table 1:** Baseline characteristics of 5,170 people living with HIV according to enrollment calendar period.

Characteristic	2007–2009 *N* = 572 (11.1%)	2010–2012 *N* = 222 (4.3%)	2013–2015 *N* = 1077 (20.8%)	2016–2018 *N* = 812 (15.7%)	2019–2021 *N* = 1,353 (26.2%)	2022–2024 *N* = 1,134 (21.9%)
Sex at birth, female, *n* (%)	186 (32.5)	53 (23.9)	277 (25.7)	208 (23.1)	312 (23.1)	294 (25.9)
Non-Caucasian, *n* (%)	43 (7.5)	15 (6.8)	72 (6.7)	77 (9.5)	162 (12.0)	121 (10.7)
Age (years), mean ± SD	45.3 ± 8.8	45.2 ± 9.6	44.9 ± 10.6	48.1 ± 11.6	48.7 ± 12.4	48.8 ± 12.4
Age category, *n* (%)						
*< 40 years*	114 (19.9)	54 (24.3)	323 (30.0)	174 (21.4)	315 (23.3)	288 (25.4)
*40–49 years*	332 (58.0)	105 (47.3)	404 (37.5)	202 (24.9)	349 (25.7)	280 (24.7)
*50–59 years*	91 (15.9)	47 (21.1)	269 (25.0)	330 (40.6)	458 (33.9)	332 (29.3)
*60–69 years*	25 (4.4)	15 (6.8)	62 (5.7)	86 (10.6)	173 (12.8)	195 (17.2)
*≥70 years*	10 (1.7)	1 (0.5)	19 (1.8)	20 (2.5)	58 (4.3)	39 (3.4)
BMI (kg/m^2^), mean ± SD	23.3 ± 3.9	24.5 ± 4.8	24.0 ± 3.6	24.3 ± 4.1	25.2 ± 4.5	25.2 ± 4.3
History of IDU, *n* (%)	216 (37.8)	81 (36.5)	195 (18.1)	172 (21.2)	156 (11.5)	131 (11.6)
HCV positive, *n* (%)	216 (38.5)	100 (47.2)	233 (22.0)	192 (24.5)	189 (15.3)	161 (14.5)
CDC stage C, *n* (%)	226 (39.5)	85 (38.3)	230 (21.4)	203 (25.0)	254 (19.4)	212 (19.3)
CD4 cells/mm^3^, median (IQR)	307 (175-470)	322 (156-547)	501 (306-732)	569 (333-796)	626 (419-864)	669 (459-936)
ART-naïve, *n (*%)	15 (2.6)	44 (19.8)	272 (25.3)	171 (21.1)	256 (18.9)	181 (16.0)
ART-experienced, *n* (%)	557 (97.4)	178 (80.2)	805 (74.7)	641 (78.9)	1,097 (81.1)	953 (84.0)
HIV RNA ≥50 copies/mL, *n* (%)	367 (65.9)	108 (60.7)	166 (20.6)	86 (13.4)	136 (12.4)	64 (6.7)
Previous ART (years), median (IQR) ^*^	11.9 (9.4–14.1)	10.5 (3.6–14.1)	8.3 (3.4–16.0)	11.1 (4.7–19.0)	9.1 (4.0–16.7)	10.9 (6.3–18.4)
Drug cohort, *n* (%)						
*RAL*	355 (62.1)	103 (46.4)	20 (1.9)	—	—	—
*DRV/r*	217 (37.9)	119 (53.6)	12 (1.1)	—	—	—
*EVG*	—	—	288 (26.7)	44 (5.4)	—	—
*RPV*	—	—	459 (42.6)	2 (0.2)	—	—
*DTG*	—	—	298 (27.7)	524 (64.5)	446 (33.0)	114 (10.0)
*DRV/c*	—	—	—	242 (29.8)	—	—
*DOR*	—	—	—	—	213 (15.7)	158 (13.9)
*BIC*	—	—	—	—	694 (51.3)	253 (22.3)
*CAB*	—	—	—	—	—	609 (53.7)
TDF in current regimen, *n* (%)	256 (44.8)	121 (54.5)	846 (78.6)	223 (27.5)	131 (9.7)	112 (9.9)
TDF in previous regimen, *n* (%)	306 (53.5)	105 (47.3)	539 (50.0)	266 (32.8)	125 (9.2)	47 (4.1)
Baseline eGFR category, *n* (%)						
*≥ 90*	327 (57.2)	108 (48.6)	626 (58.1)	442 (54.4)	630 (46.6)	507 (44.7)
*60– < 90*	215 (37.6)	93 (41.9)	419 (38.9)	329 (40.5)	622 (46.0)	547 (48.2)
*30– < 60*	30 (5.2)	21 (9.5)	32 (3.0)	41 (5.1)	101 (7.5)	80 (7.0)

Values are shown as n (%), mean ± SD, or median (IQR), as appropriate. ^*^Previous ART duration refers to ART-experienced participants. HIV RNA percentages are based on available data (*N* = 4,253). BMI, body mass index; IDU, injection drug user; CDC, center for disease control and prevention; IQR, interquartile range; ART, antiretroviral therapy; RAL, raltegravir; DRV/r, darunavir/ritonavir; EVG, elvitegravir; RPV, rilpivirine; DTG, dolutegravir; DRV/c, darunavir/cobicistat; DOR, doravirine; BIC, bictegravir; CAB, cabotegravir; TDF, tenofovir disoproxil fumarate; eGFR, estimated glomerular filtration rate.

Among ART-naïve participants, baseline eGFR increased across calendar periods, from 94 mL/min/1.73 m^2^ in 2007–2009 to 107 mL/min/1.73 m^2^ from 2016–2018 onward (linear trend *p* = 0.0006), despite no significant change in mean age over time. In contrast, among ART-experienced participants, mean age increased over time from 45.4 to 50.0 years, while mean baseline eGFR declined from 99 to 87 mL/min/1.73 m^2^ (linear trend *p* < 0.0001).

### Changes in eGFR from baseline to week 24

**Figure 1:**
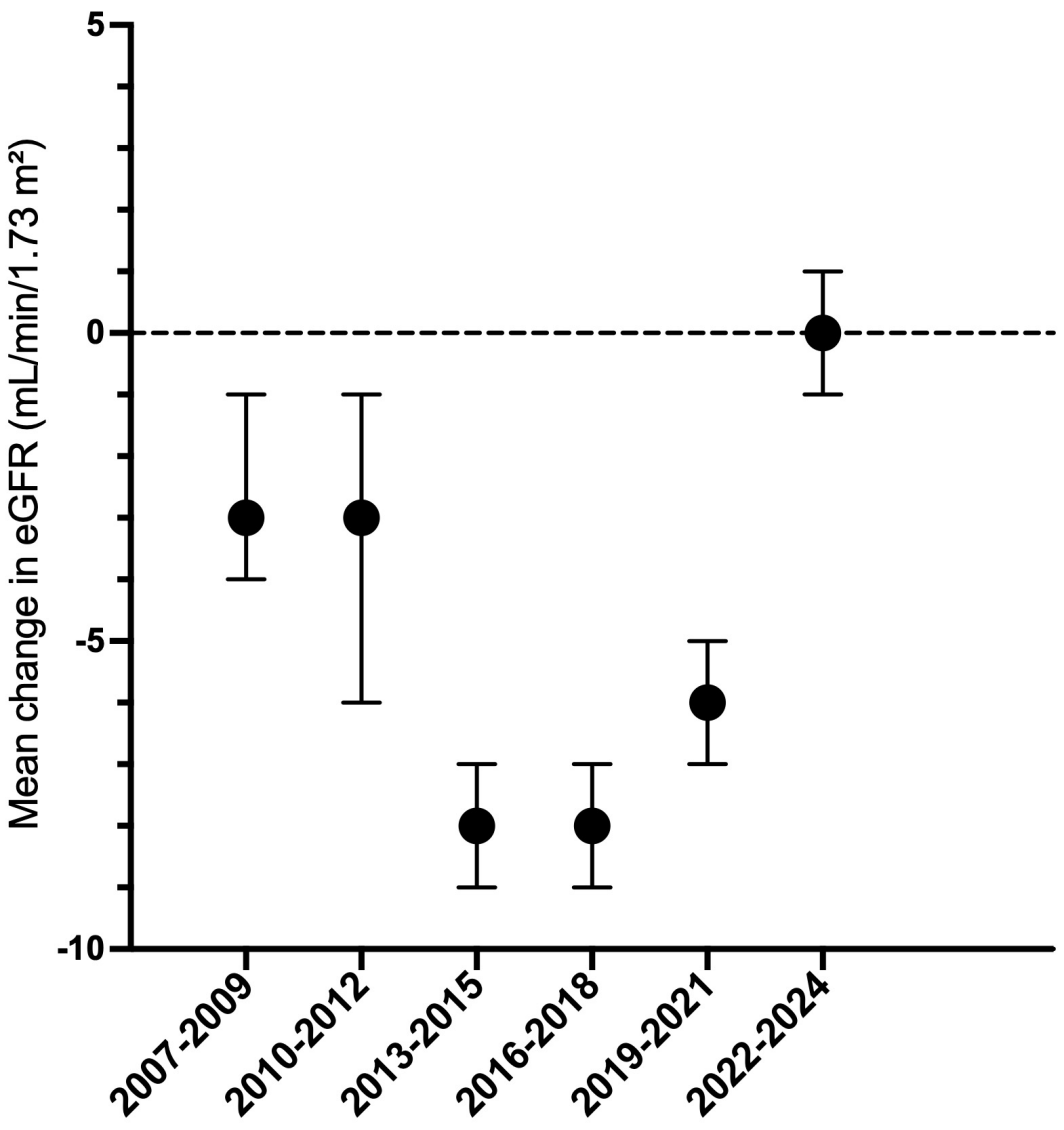
Mean unadjusted eGFR change from baseline to week 24 across calendar periods. Points represent mean values and error bars 95% confidence intervals.

Changes in eGFR from baseline to week 24 are reported in Table 2, while adjusted model estimates are shown in Supplementary Tables S2A, S2B. The main decline in eGFR occurred during the first 24 weeks after ART initiation or switch (Figure 1).

**Table 2:** Unadjusted change in eGFR from baseline to W24 according to enrollment calendar period and subgroup.

Subgroup	2007–2009 *N* = 572 (11.1%)	2010–2012 *N* = 222 (4.3%)	2013–2015 *N* = 1,077 (20.8%)	2016–2018 *N* = 812 (15.7%)	2019–2021 *N* = 1,353 (26.2%)	2022–2024 *N* = 1,134 (21.9%)
Total	−3 (−4 to −1)	−3 (−6 to −1)	−8 (−9 to −7)	−8 (−9 to −7)	−6 (−7 to −5)	0 (−1 to 1)
Sex						
*Women*	−5 (−7 to −2)	−3 (−7 to 0)	−7 (−9 to −5)	−7 (−9 to −5)	−6 (−8 to −4)	1 (−1 to 3)
*Men*	−2 (−4 to 0)	−4 (−6 to −1)	−9 (−10 to −8)	−8 (−9 to −6)	−5 (−7 to −4)	0 (−2 to 1)
Ethnicity						
*Caucasian*	−3 (−5 to −2)	−3 (−6 to −1)	−8 (−9 to −7)	−8 (−9 to −6)	−5 (−6 to −4)	1 (0 to 2)
*Non–Caucasian*	2 (−3 to 7)	−5 (−15 to 4)	−8 (−11 to −4)	−8 (−13 to −4)	−8 (−11 to −5)	−5 (−9 to 0)
Age category						
*< 40 years*	−2 (−5 to 2)	−2 (−8 to 4)	−9 (−11 to −8)	−12 (−14 to −9)	−8 (−10 to −6)	−1 (−4 to 2)
*40–49 years*	−4 (−6 to −3)	−3 (−5 to 0)	−7 (−9 to −6)	−6 (−8 to −4)	−7 (−10 to −5)	−1 (−4 to 2)
*50–59 years*	0 (−3 to 3)	−6 (−12 to 0)	−8 (−10 to −6)	−6 (−8 to −5)	−3 (−5 to −2)	1 (−1 to 2)
*60–69 years*	−1 (−6 to 4)	−8 (−15 to 2)	−10 (−14 to −6)	−9 (−12 to −6)	−5 (−7 to −2)	3 (0 to 5)
*≥70 years*	−3 (−15 to 9)	—	−9 (−14 to −4)	−7 (−15 to 0)	−1 (−4 to 2)	−2 (−5 to 1)
BMI (*n* = 4,535)						
*< 25 kg/m^2^*	−2 (−4 to −1)	−4 (−7 to −2)	−8 (−10 to −7)	−9 (−11 to −7)	−6 (−8 to −5)	0 (−2 to 1)
*≥25 kg/m^2^*	−3 (−5 to 0)	−2 (−6 to 3)	−9 (−10 to −7)	−6 (−7 to −4)	−4 (−5 to −3)	1 (−1 to 2)
History of IDU						
*No*	−2 (−4 to 0)	−5 (−8 to −1)	−9 (−10 to −8)	−8 (−9 to −7)	−6 (−7 to −5)	0 (−1 to 1)
*Yes*	−4 (−7 to −2)	−2 (−4 to 1)	−5 (−7 to −3)	−7 (−10 to −5)	−3 (−6 to −1)	1 (−2 to 3)
CDC'93 classification stage						
*A/B*	−3 (−4 to −1)	−4 (−7 to −1)	−8 (−9 to −7)	−7 (−9 to −6)	−6 (−7 to −5)	0 (−1 to 2)
*C*	−3 (−5 to −1)	−3 (−7 to 2)	−9 (−11 to −7)	−9 (−11 to −6)	−5 (−8 to −3)	−1 (−4 to 1)
Current TDF–containing regimen						
*No*	−1 (−3 to 1)	−4 (−7 to −1)	−8 (−10 to −6)	−7 (−9 to −6)	−6 (−7 to −5)	0 (−1 to 2)
*Yes*	−5 (−7 to −3)	−3 (−7 to 0)	−8 (−9 to −7)	−9 (−11 to −7)	−3 (−5 to 0)	1 (−5 to 2)
ART–naïve	−3 (−9 to 4)	−6 (−13 to 1)	−11 (−13 to −9)	−15 (−19 to −12)	−15 (−17 to −12)	−13 (−17 to −10)
ART–experienced	−3 (−4 to −1)	−3 (−5 to 0)	−7 (−8 to −6)	−6 (−7 to −5)	−3 (−4 to −2)	3 (2 to 4)
Current drug cohort						
*RAL*	−2 (−4 to 0)	−2 (−5 to 1)	−4 (−10 to 1)	—	—	—
*DRV/r*	−4 (−6 to −2)	−5 (−8 to −1)	−1 (−8 to 5)	—	—	—
*EVG*	—	—	−10 (−12 to −8)	−7 (−12 to −2)	—	—
*RPV*	—	—	−6 (−8 to −5)	—	—	—
*DTG*	—	—	−11 (−12 to −9)	−9 (−10 to −7)	−9 (−12 to −7)	−7 (−11 to −3)
*DRV/c*	—	—	—	−6 (−8 to −4)	—	—
*DOR*	—	—	—	—	−2 (−4 to 0)	−2 (−5 to 1)
*BIC*	—	—	—	—	−4 (−5 to −3)	−9 (−12 to −7)
*CAB*	—	—	—	—	—	6 (5 to 7)

Values are unadjusted mean change in eGFR (95% CI), expressed as mL/min/1.73 m^2^. Negative values indicate a decline in eGFR. Column headers report the number of participants enrolled in each calendar period; subgroup estimates are based on available eGFR measurements at the relevant follow-up timepoint. BMI, body mass index; IDU, injection drug user; CDC, center for disease control and prevention; IQR, interquartile range; ART, antiretroviral therapy; RAL, raltegravir; DRV/r, darunavir/ritonavir; EVG, elvitegravir; RPV, rilpivirine; DTG, dolutegravir; DRV/c, darunavir/cobicistat; DOR, doravirine; BIC, bictegravir; CAB, cabotegravir; TDF, tenofovir disoproxil fumarate; eGFR, estimated glomerular filtration rate.

Mean unadjusted changes from baseline to week 24 were modest in 2007–2009 and 2010–2012 (−3 mL/min/1.73 m^2^ in both periods), more pronounced in 2013–2015 and 2016–2018 (−8 mL/min/1.73 m^2^ in both periods), smaller in 2019–2021 (−5 mL/min/1.73 m^2^), and negligible in 2022–2024 (0 mL/min/1.73 m^2^). In both multivariable models, ART-naïve participants showed a greater decline in eGFR than ART-experienced individuals (adjusted mean change −6.21 to −7.60 vs. −3.27 to −3.29 mL/min/1.73 m^2^; *p* < 0.0001). The association between TDF use and early eGFR changes was not consistent across the two models. In the model including drug cohort, adjusted mean changes were virtually identical in TDF-containing and non-TDF-containing regimens (−4.75 vs. −4.75 mL/min/1.73 m^2^; *p* = 0.9935), whereas in the model including calendar period, TDF-containing regimens showed a slightly smaller decline (−4.61 vs. −6.27 mL/min/1.73 m^2^; *p* = 0.0031). Cohort- and period-specific adjusted estimates are reported in Supplementary Tables S2A, S2B. In both models, the decline in eGFR appeared attenuated in the most recent strata.

### Changes in eGFR from week 24 to week 48

Unadjusted changes in eGFR from week 24 to week 48 are reported in Supplementary Table S3, whereas adjusted models are shown in Supplementary Tables S4A, S4B. Changes in eGFR from T1 to T2 were minimal across all calendar periods: 0 mL/min/1.73 m^2^ (95% CI −2 to 1) in 2007–2009, 1 mL/min/1.73 m^2^ (95% CI −2 to 4) in 2010–2012, 0 mL/min/1.73 m^2^ (95% CI −2 to 1) in 2013–2015, −1 mL/min/1.73 m^2^ (95% CI −2 to 1) in 2016–2018, 0 mL/min/1.73 m^2^ (95% CI −1 to 1) in 2019–2021, and 0 mL/min/1.73 m^2^ (95% CI −1 to 1) in 2022–2024. No linear trend over time was observed (*p* = 0.97), and no significant differences emerged across calendar periods or treatment cohorts. In unadjusted analyses, no significant differences in T1-to-T2 eGFR changes were observed according to sex, age, ethnicity, BMI, history of IDU, CDC stage, ART-naïve status, or TDF use in the current regimen. In multivariable analyses, only baseline eGFR remained modestly associated with T1-to-T2 changes (β approximately −0.024 to −0.025; *p* = 0.02).

### Kidney-related treatment discontinuation

Kidney-related treatment discontinuations were assessed over the entire available follow-up and were uncommon. Over a median follow-up time of 24 months (interquartile range, 13–40), 26 discontinuations were recorded overall (0.5%), including 22 due to kidney adverse events and 4 due to treatment changes intended to preserve renal function. These events were more frequent among participants receiving TDF-containing regimens than among those receiving other regimens (18/1689 [1.0%] vs. 8/3481 [0.2%], *p* < 0.001).

## Discussion

In this large observational cohort, severe renal impairment at enrollment was uncommon, but the renal profile of participants changed substantially over time. This change did not reflect an increase in advanced CKD; rather, it was driven by a progressive shift in case-mix, with older age, lower prevalence of IDU and participants with positive HCV serology, and a higher proportion of ART-experienced participants in recent years. The main decline in eGFR occurred during the first follow-up interval after ART initiation or switch, while changes from T1 to T2 were minimal. Overall, these findings suggest that contemporary renal changes in PWH are driven more by aging, treatment history, regimen selection, and creatinine-handling effects than by progressive nephrotoxicity alone (14, 15).

The baseline pattern observed in our cohort reflects the current profile of kidney disease in PWH, which is now influenced more by aging, comorbidities, and cumulative treatment exposure than by classic HIV-associated nephropathy alone. Recent reviews and consensus documents have emphasized that, in the ART era, CKD in PWH is increasingly associated with aging, hypertension, diabetes, obesity, positive HCV serology, and long-term treatment exposure, while the spectrum of renal disease has shifted away from HIVAN as the dominant phenotype (1–3, 5). In this context, the lower baseline eGFR observed in participants with BMI ≥25 kg/m^2^ and in ART-experienced individuals is biologically plausible and consistent with the growing burden of metabolic and age-related renal risk factors in aging HIV cohorts (5, 16). This pattern also fits with the emerging cardiovascular-kidney-metabolic framework in PWH, in which adiposity, chronic inflammation, metabolic dysfunction, and renal risk increasingly converge (7, 17). Mean age also increased over time among ART-experienced participants, together with a decline in baseline eGFR, consistent with the expected age-related reduction in kidney function observed in the general population (18).

Among ART-naïve individuals, baseline eGFR increased across calendar periods despite stable mean age. This pattern likely reflects changes in HIV care over time, including earlier treatment initiation and less advanced disease at ART start in more recent years, rather than a true improvement in intrinsic renal health. In adjusted analyses, the early decline in eGFR from T0 to T1 was greater among ART-naïve than among ART-experienced participants. This may partly reflect the substantially higher baseline eGFR observed in naïve individuals, with a larger early downward shift after treatment initiation. More broadly, creatinine-based eGFR may capture early changes related to treatment initiation and creatinine handling rather than structural renal injury (1, 10, 19). The absence of clinically meaningful change between T1 and T2 also suggests that progressive short-term nephrotoxicity was unlikely in most participants.

The regimen-specific findings also require cautious interpretation. Participants receiving TDF-containing regimens had higher baseline eGFR, suggesting channeling of individuals with better renal function toward regimens perceived as potentially nephrotoxic. This is consistent with cohort data showing that both cumulative and current exposure to nephrotoxic antiretrovirals, including TDF and ritonavir-boosted protease inhibitors, may contribute to CKD risk while also influencing prescribing choices in routine practice (8, 9). In our study, the association between TDF use and early eGFR change was not consistent across the two alternative adjusted models, depending on whether drug cohort or calendar period was included. This discrepancy likely reflects the non-random distribution of TDF use across calendar periods and drug cohorts in SCOLTA. TDF-containing regimens were substantially more common in earlier years, whereas their use declined markedly in more recent periods. Therefore, adjustment for drug cohort and adjustment for calendar period capture partially overlapping but not identical treatment structures, which may explain the different TDF estimates across models. These data therefore do not support a simple causal interpretation of early eGFR decline according to TDF exposure alone. Kidney-related treatment discontinuations remained uncommon overall, although they were more frequent in participants receiving TDF-containing regimens. This finding is broadly consistent with earlier SCOLTA observations, in which clinically overt TDF-related renal toxicity was uncommon in routine practice despite persistent concern regarding renal safety (20).

The attenuation of early eGFR decline in the most recent period should be interpreted cautiously. This pattern may partly reflect the increasing use of contemporary switch strategies, including CAB-based regimens, but it may also be related to broader changes in regimen mix and patient selection over time. Because calendar period and drug cohort partially overlapped by design, these effects are difficult to separate in an observational pharmacovigilance cohort such as SCOLTA (12). In settings where INSTI-based switch strategies are increasingly used, small early creatinine changes may reflect altered tubular handling rather than true reductions in glomerular filtration (10, 11).

This study has several strengths, including its large sample size, multicenter real-world setting, and long enrollment period spanning major changes in HIV treatment practice. However, its observational design leaves room for confounding by indication, channeling bias, and residual confounding, as several relevant determinants of renal function, including hypertension, diabetes, cardiovascular disease, smoking, proteinuria, and nephrotoxic non-ART medications, were not uniformly available for adjustment. Renal assessment relied on creatinine-based eGFR, which may partly reflect altered creatinine handling rather than true changes in filtration. The use of the 2009 CKD-EPI equation may also have affected absolute eGFR estimates, although the study focused primarily on within-person changes. Findings apply only to participants with baseline eGFR ≥30 mL/min/1.73 m^2^ and should not be extrapolated to those with severe renal dysfunction. Analyses were limited to prespecified 24- and 48-week windows and therefore do not capture longer-term renal trajectories; beyond 48 weeks, treatment changes, unequal follow-up, and attrition would have increased heterogeneity. Finally, calendar period and drug cohort were closely intertwined, limiting causal interpretation of temporal and regimen-specific differences.

In conclusion, in this large Italian HIV cohort restricted to participants with baseline eGFR ≥30 mL/min/1.73 m^2^, severe renal impairment at enrollment and kidney-related treatment discontinuations were uncommon. However, the baseline renal profile of participants changed over time as the cohort became older and more ART-experienced. The main decline in creatinine-based eGFR occurred during the first 24 weeks after ART initiation or switch, was greater among ART-naïve individuals, and was followed by relative stability between 24 and 48 weeks. These findings support the overall renal tolerability of contemporary ART in routine clinical practice among participants with baseline eGFR ≥30 mL/min/1.73 m^2^, while also underscoring the need for continued monitoring as aging, metabolic risk factors, and cumulative treatment exposure increasingly shape kidney health in PWH (3, 5).

## Group member of CISAI Study Group

E. Sarchi, C. Bolla (Alessandria); A. Saracino, D. Fiordelisi (Bari); L. Calza (Bologna); B. Menzaghi, M. Farinazzo, F. Franzetti (Busto Arsizio); G. Angioni, M. Buffa (Cagliari); A. Masiello, F. Simeone (Caserta); G. Nunnari, B. M. Celesia (Catania); K. Falasca, M. Pontolillo (Chieti); L. Pusterla, G. Chieffo (Como); A. Mastroianni, G. Guadagnino (Cosenza); P. Maggi (Enna); E. Salomoni, L. Attala (Firenze); F. Lagi, S. Trevisan (Firenze); S. Ferrara, C. Grillo (Foggia); A. Di Biagio, S. Blanchi, L. Taramasso, M. Bassetti (Genova); E. Pontali, A. Parisini, F. Del Puente (Genova); C. Molteni, A. Pandolfo, S. Piconi (Lecco); S. Rusconi, M. Franzetti (Legnano); G. F. Pellicanò (Messina); Bandera (Milano); D. Motta, M. Merli, M. Puoti (Milano); T. Bini (Milano); P. Bonfanti, N. Squillace, V. Cogliandro (Monza); S. Martini (Napoli); V. Esposito, M.A. Carleo, S. Mascolo (Napoli); O. Bargiacchi, P.L. Garavelli (Novara); A. Cascio, M. Trizzino (Palermo); R. Gulminetti, L. Pagnucco (Pavia); G. V. De Socio, A. Gidari, I. Antinoro (Perugia); S. Cicalini, R. Bellagamba, A. Antinori (Roma); P. Morelli (Rozzano, Humanitas); G. Cenderello, V. Chinni (Sanremo); G. Madeddu, A. De Vito (Sassari); S. Grignolo (Savona); G. Orofino, M. Guastavigna (Torino); M. Lanzafame, A. Delama (Trento); A. Londero, A. Giacinta, C. Tascini (Udine); G. Battagin, V. Manfrin (Vicenza).

## Data Availability

The datasets presented in this article are not readily available because the data that support the findings of this study are not publicly available because they contain information that could compromise participant privacy. Data may be made available from the corresponding author upon reasonable request and with appropriate approvals. Requests to access the datasets should be directed to Gruppo C.I.S.A.I., https://cisai.it/Contatti.aspx.
