# Supplementary material for: Changing renal profile and early eGFR dynamics after ART initiation or switch in a large Italian HIV cohort
Source: Front Med (Lausanne). 2026 Aug 11;13:1898349. doi: 10.3389/fmed.2026.1898349 (PMC13503182; doi:10.3389/fmed.2026.1898349)
Supplement: Supplementary file 1 [file Table_1.docx]

**SUPPLEMENTARY TABLES**

**Supplementary Table S1. Baseline eGFR (mL/min/1.73 m²) according to enrollment calendar period and subgroup.**

| **Subgroup** | **2007-2009 N=572 (11.1%)** | **2010-2012 N=222 (4.3%)** | **2013-2015 N=1077 (20.8%)** | **2016-2018 N=812 (15.7%)** | **2019-2021 N=1353 (26.2%)** | **2022-2024 N=1134 (21.9%)** |
| --- | --- | --- | --- | --- | --- | --- |
|  |  |  |  |  |  |  |
| **Total** | **99 ± 27** | **90 ± 25** | **97 ± 23** | **95 ± 24** | **91 ± 26** | **91 ± 26** |
| Sex |  |  |  |  |  |  |
| *Women* | 96 ± 26 | 90 ± 30 | 93 ± 23 | 90 ± 23 | 89 ± 28 | 90 ± 26 |
| *Men* | 100 ± 27 | 90 ± 24 | 98 ± 22 | 96 ± 25 | 91 ± 25 | 91 ± 26 |
| Ethnicity |  |  |  |  |  |  |
| *Caucasian* | 98 ± 26 | 89 ± 24 | 96 ± 22 | 94 ± 24 | 89 ± 25 | 88 ± 24 |
| *Non-Caucasian* | 105 ± 29 | 102 ± 37 | 109 ± 25 | 102 ± 26 | 105 ± 27 | 109 ± 33 |
| Age category |  |  |  |  |  |  |
| *<40 years* | 103 ± 28 | 100 ± 31 | 105 ± 22 | 109 ± 26 | 105 ± 25 | 105 ± 27 |
| *40-49 years* | 101 ± 25 | 88 ± 23 | 96 ± 22 | 95 ± 22 | 96 ± 26 | 92 ± 28 |
| *50-59 years* | 96 ± 24 | 88 ± 21 | 92 ± 21 | 90 ± 22 | 85 ± 24 | 86 ± 19 |
| *60-69 years* | 70 ± 22 | 81 ± 26 | 85 ± 22 | 85 ± 22 | 79 ± 20 | 79 ± 20 |
| *≥70 years* | 76 ± 25 | — | 74 ± 22 | 76 ± 25 | 70 ± 18 | 71 ± 17 |
| BMI (n=4535) |  |  |  |  |  |  |
| *<25 kg/m²* | 99 ± 26 | 92 ± 28 | 97 ± 23 | 96 ± 24 | 92 ± 27 | 92 ± 26 |
| *≥25 kg/m²* | 89 ± 24 | 87 ± 23 | 96 ± 23 | 89 ± 22 | 88 ± 23 | 90 ± 25 |
| History of IDU |  |  |  |  |  |  |
| *No* | 101 ± 26 | 89 ± 23 | 96 ± 22 | 95 ± 25 | 89 ± 23 | 88 ± 20 |
| *Yes* | 97 ± 27 | 91 ± 27 | 97 ± 23 | 94 ± 24 | 91 ± 26 | 91 ± 26 |
| CDC ’93 classification stage |  |  |  |  |  |  |
| *A/B* | 96 ± 26 | 91 ± 26 | 97 ± 22 | 94 ± 23 | 92 ± 25 | 91 ± 26 |
| *C* | 102 ± 27 | 88 ± 25 | 97 ± 27 | 95 ± 27 | 90 ± 27 | 89 ± 26 |
| ART-naïve | 94 ± 24 | 99 ± 33 | 104 ± 23 | 107 ± 28 | 108 ± 27 | 107 ± 29 |
| ART-experienced | 99 ± 27 | 88 ± 23 | 95 ± 22 | 91 ± 22 | 87 ± 24 | 87 ± 24 |
| TDF in previous regimen (ART-experienced) |  |  |  |  |  |  |
| *No* | 101 ± 29 | 85 ± 25 | 93 ± 23 | 91 ± 23 | 87 ± 24 | 98 ± 23 |
| *Yes* | 97 ± 25 | 90 ± 20 | 95 ± 22 | 92 ± 21 | 92 ± 23 | 102 ± 37 |
| Current TDF-containing regimen |  |  |  |  |  |  |
| *No* | 97 ± 28 | 84 ± 22 | 91 ± 23 | 93 ± 24 | 91 ± 26 | 90 ± 25 |
| *Yes* | 100 ± 25 | 96 ± 27 | 99 ± 22 | 100 ± 24 | 94 ± 21 | 97 ± 29 |
| Current drug cohort |  |  |  |  |  |  |
| *RAL* | 97 ± 26 | 86 ± 24 | 83 ± 24 | — | — | — |
| *DRV/r* | 102 ± 27 | 94 ± 26 | 95 ± 23 | — | — | — |
| *EVG* | — | — | 99 ± 24 | 102 ± 19 | — | — |
| *RPV* | — | — | 98 ± 21 | 114 ± 2 | — | — |
| *DTG* | — | — | 94 ± 23 | 93 ± 25 | 95 ± 28 | 91 ± 26 |
| *DRV/c* | — | — | — | 96 ± 23 | — | — |
| *DOR* | — | — | — | — | 90 ± 23 | 92 ± 29 |
| *BIC* | — | — | — | — | 89 ± 25 | 101 ± 29 |
| *CAB* | — | — | — | — | — | 86 ± 21 |

*Values are mean ± SD. Abbreviations: BMI, body mass index; IDU, injection drug user; CDC, center for disease control and prevention; IQR, interquartile range; ART, antiretroviral therapy; RAL, raltegravir; DRV/r, darunavir/ritonavir; EVG, elvitegravir; RPV, rilpivirine; DTG, dolutegravir; DRV/c, darunavir/cobicistat; DOR, doravirine; BIC, bictegravir; CAB, cabotegravir; TDF, tenofovir disoproxil fumarate; eGFR, estimated Glomerular Filtration Rate.*

**Supplementary Table S2A. Adjusted model for change in eGFR from baseline to W24 including drug cohort.**

| **Effect** | **Level / contrast** | **Adjusted mean change (95% CI)** | **p value** |
| --- | --- | --- | --- |
| Baseline eGFR | Per 1 mL/min/1.73 m² increase | β = -0.337 | **<0.0001** |
| ART status | ART-experienced | -3.29 (-3.87 to -2.72) | **<0.0001** |
|  | ART-naïve | -6.21 (-7.33 to -5.08) |  |
| Current TDF use | No TDF | -4.75 (-5.68 to -3.82) | 0.9935 |
|  | TDF-containing | -4.75 (-5.69 to -3.81) |  |
| BMI category | Overall effect | — | 0.1099 |
| Drug cohort | Overall effect | — | **<0.0001** |
|  | RAL | -2.95 (-4.45 to -1.46) |  |
|  | DRV/r | -3.26 (-4.95 to -1.57) |  |
|  | EVG | -7.79 (-9.60 to -5.98) |  |
|  | RPV | -4.80 (-6.37 to -3.22) |  |
|  | DTG | -9.43 (-10.43 to -8.43) |  |
|  | DRV/c | -6.45 (-8.44 to -4.46) |  |
|  | DOR | -3.90 (-5.54 to -2.26) |  |
|  | BIC | -6.45 (-7.64 to -5.26) |  |
|  | CAB | 2.30 (0.82 to 3.77) |  |

*Adjusted means and p values are taken from the SAS output. The model included drug cohort, ART-naïve status, BMI category, TDF use in the current regimen, and baseline eGFR. Because eGFR was calculated with the 2009 CKD-EPI equation, age, sex, and ethnicity were embedded in the outcome and were not entered separately as model covariates. Abbreviations: BMI, body mass index; ART, antiretroviral therapy; RAL, raltegravir; DRV/r, darunavir/ritonavir; EVG, elvitegravir; RPV, rilpivirine; DTG, dolutegravir; DRV/c, darunavir/cobicistat; DOR, doravirine; BIC, bictegravir; CAB, cabotegravir; TDF, tenofovir disoproxil fumarate; eGFR, estimated Glomerular Filtration Rate*

**Supplementary Table S2B. Adjusted model for change in eGFR from baseline to W24 including calendar period.**

| **Effect** | **Level / contrast** | **Adjusted mean change (95% CI)** | **p value** |
| --- | --- | --- | --- |
| Baseline eGFR | Per 1 mL/min/1.73 m² increase | β = -0.344 | **<0.0001** |
| ART status | ART-experienced | -3.27 (-3.90 to -2.65) | **<0.0001** |
|  | ART-naïve | -7.60 (-8.71 to -6.49) |  |
| Current TDF use | No TDF | -6.27 (-7.08 to -5.45) | **0.0031** |
|  | TDF-containing | -4.61 (-5.56 to -3.66) |  |
| BMI category | Overall effect | — | **0.0474** |
| Calendar period | Overall effect | — | **<0.0001** |
|  | 2007-2009 | -2.68 (-4.10 to -1.26) |  |
|  | 2010-2012 | -5.65 (-7.71 to -3.58) |  |
|  | 2013-2015 | -8.34 (-9.41 to -7.27) |  |
|  | 2016-2018 | -7.96 (-9.12 to -6.80) |  |
|  | 2019-2021 | -6.69 (-7.70 to -5.69) |  |
|  | 2022-2024 | -1.31 (-2.41 to -0.21) |  |

*Adjusted means and p values are taken from the SAS output. The model included calendar period, ART-naïve status, BMI category, TDF use in the current regimen, and baseline eGFR. Because eGFR was calculated with the 2009 CKD-EPI equation, age, sex, and ethnicity were embedded in the outcome and were not entered separately as model covariates. Abbreviations: BMI, body mass index; eGFR, estimated Glomerular Filtration Rate*

**Supplementary Table S3. Unadjusted change in eGFR from W24 to W48 according to enrollment calendar period and subgroup.**

| **Subgroup** | **2007-2009 N=572 (11.1%)** | **2010-2012 N=222 (4.3%)** | **2013-2015 N=1077 (20.8%)** | **2016-2018 N=812 (15.7%)** | **2019-2021 N=1353 (26.2%)** | **2022-2024 N=1134 (21.9%)** |
| --- | --- | --- | --- | --- | --- | --- |
|  |  |  |  |  |  |  |
| **Total** | **0 (-2 to 1)** | **1 (-2 to 4)** | **0 (-2 to 1)** | **-1 (-2 to 1)** | **0 (-1 to 1)** | **0 (-1 to 1)** |
| Sex |  |  |  |  |  |  |
| *Women* | -1 (-3 to 2) | 4 (-6 to 13) | 1 (-2 to 3) | -1 (-4 to 1) | 0 (-2 to 2) | -2 (-4 to 0) |
| *Men* | 0 (-2 to 2) | 0 (-2 to 2) | -1 (-2 to 0) | 0 (-2 to 1) | 0 (-1 to 1) | 0 (-1 to 2) |
| Ethnicity |  |  |  |  |  |  |
| *Caucasian* | 0 (-2 to 1) | 0 (-2 to 2) | 0 (-1 to 1) | 0 (-2 to 1) | 0 (-1 to 1) | 0 (-1 to 1) |
| *Non-Caucasian* | 4 (-3 to 11) | 12 (-24 to 47) | -4 (-9 to 2) | -2 (-7 to 2) | -2 (-5 to 1) | -3 (-7 to 0) |
| Age category |  |  |  |  |  |  |
| *<40 years* | 1 (-3 to 4) | 5 (-5 to 15) | -2 (-3 to 0) | 3 (-3 to 8) | -1 (-3 to 2) | -1 (-4 to 2) |
| *40-49 years* | -1 (-3 to 1) | -1 (-4 to 2) | 1 (-2 to 3) | -1 (-4 to 2) | 0 (-1 to 1) | 0 (-2 to 2) |
| *50-59 years* | 1 (-4 to 5) | 0 (-4 to 4) | -1 (-2 to 1) | -2 (-3 to 0) | 0 (-1 to 2) | 1 (-1 to 3) |
| *60-69 years* | 5 (0 to 9) | -1 (-4 to 2) | -1 (-4 to 2) | -1 (-4 to 2) | -1 (-3 to 2) | -2 (-4 to 1) |
| *≥70 years* | -5 (-13 to 3) | 6 (— to —) | 2 (-3 to 7) | 1 (-4 to 6) | 0 (-2 to 2) | 0 (-5 to 4) |
| BMI (n=4535) |  |  |  |  |  |  |
| *<25 kg/m²* | 0 (-2 to 2) | 1 (-4 to 6) | 0 (-2 to 1) | -1 (-2 to 1) | 0 (-1 to 1) | 0 (-2 to 2) |
| *≥25 kg/m²* | 2 (0 to 5) | -1 (-3 to 1) | 0 (-2 to 3) | -2 (-4 to 0) | 0 (-2 to 1) | 0 (-1 to 1) |
| History of IDU |  |  |  |  |  |  |
| *No* | 1 (-1 to 3) | 1 (-3 to 6) | -1 (-2 to 0) | -1 (-2 to 1) | 0 (-1 to 1) | 0 (-2 to 1) |
| *Yes* | -2 (-5 to 0) | 0 (-2 to 3) | 1 (-1 to 3) | -1 (-3 to 2) | -2 (-5 to 1) | 1 (-1 to 3) |
| CDC ’93 classification stage |  |  |  |  |  |  |
| *A/B* | 0 (-2 to 1) | 1 (-3 to 5) | 0 (-2 to 1) | -1 (-2 to 1) | -1 (-1 to 0) | 0 (-1 to 1) |
| *C* | 0 (-3 to 3) | 0 (-4 to 4) | -1 (-3 to 1) | 0 (-3 to 3) | 1 (-1 to 4) | -2 (-4 to 0) |
| Current TDF-containing regimen |  |  |  |  |  |  |
| *No* | 0 (-2 to 2) | 0 (-2 to 3) | 1 (-2 to 3) | -1 (-2 to 1) | 0 (-1 to 1) | 0 (-1 to 1) |
| *Yes* | 0 (-2 to 2) | 1 (-4 to 6) | -1 (-2 to 0) | -1 (-3 to 2) | -3 (-6 to 0) | 0 (-3 to 4) |
| ART-naïve | 1 (-2 to 5) | 3 (-10 to 15) | -1 (-3 to 0) | 3 (-3 to 8) | -1 (-3 to 1) | 0 (-4 to 5) |
| ART-experienced | 0 (-2 to 1) | 0 (-2 to 3) | 0 (-1 to 1) | -2 (-3 to 0) | 0 (-1 to 1) | 0 (-1 to 1) |
| Drug cohort |  |  |  |  |  |  |
| *RAL* | -1 (-2 to 1) | -1 (-3 to 1) | 1 (-5 to 7) | — | — | — |
| *DRV/r* | 1 (-2 to 3) | 3 (-2 to 8) | 2 (-10 to 14) | — | — | — |
| *EVG* | — | — | 0 (-2 to 2) | 1 (-2 to 4) | — | — |
| *RPV* | — | — | -2 (-3 to 0) | — | — | — |
| *DTG* | — | — | 1 (-1 to 3) | 0 (-2 to 2) | 0 (-1 to 2) | 1 (-2 to 4) |
| *DRV/c* | — | — | — | -2 (-4 to 0) | — | — |
| *DOR* | — | — | — | — | -2 (-4 to 1) | 1 (-2 to 3) |
| *BIC* | — | — | — | — | 0 (-1 to 1) | -1 (-4 to 3) |
| *CAB* | — | — | — | — | — | -1 (-2 to 1) |

*Values are unadjusted mean change in eGFR (95% CI), expressed as mL/min/1.73 m². Negative values indicate a decline in eGFR. “—” indicates insufficient data or not applicable for that period. Column headers report the number of participants enrolled in each calendar period; subgroup estimates are based on available eGFR measurements at the relevant follow-up timepoint. Abbreviations: BMI, body mass index; IDU, injection drug user; CDC, center for disease control and prevention; IQR, interquartile range; ART, antiretroviral therapy; RAL, raltegravir; DRV/r, darunavir/ritonavir; EVG, elvitegravir; RPV, rilpivirine; DTG, dolutegravir; DRV/c, darunavir/cobicistat; DOR, doravirine; BIC, bictegravir; CAB, cabotegravir; TDF, tenofovir disoproxil fumarate; eGFR, estimated Glomerular Filtration Rate*

**Supplementary Table S4A. Adjusted model for change in eGFR from W24 to W48 including drug cohort.**

| **Effect** | **Level / contrast** | **Adjusted mean change (95% CI)** | **p value** |
| --- | --- | --- | --- |
| Baseline eGFR | Per 1 mL/min/1.73 m² increase | β = -0.025 | **0.0201** |
| ART status | ART-experienced | -0.68 | 0.3345 |
|  | ART-naïve | 0.01 |  |
| Current TDF use | No TDF | -0.23 | 0.7954 |
|  | TDF-containing | -0.44 |  |
| BMI category | Overall effect | — | 0.8739 |
| Drug cohort | Overall effect | — | 0.2910 |

*For T1-to-T2, the overall drug cohort effect was not significant; therefore, cohort-specific least-squares means are not shown. Abbreviations: BMI, body mass index; IDU, injection drug user; CDC, center for disease control and prevention; IQR, interquartile range; ART, antiretroviral therapy; RAL, raltegravir; DRV/r, darunavir/ritonavir; EVG, elvitegravir; RPV, rilpivirine; DTG, dolutegravir; DRV/c, darunavir/cobicistat; DOR, doravirine; BIC, bictegravir; CAB, cabotegravir; TDF, tenofovir disoproxil fumarate; eGFR, estimated Glomerular Filtration Rate*

**Supplementary Table S4B. Adjusted model for change in eGFR from W24 to W48 including calendar period.**

| **Effect** | **Level / contrast** | **Adjusted mean change (95% CI)** | **p value** |
| --- | --- | --- | --- |
| Baseline eGFR | Per 1 mL/min/1.73 m² increase | β = -0.024 | **0.0241** |
| ART status | ART-experienced | -0.45 (-1.21 to 0.31) | 0.2235 |
|  | ART-naïve | 0.40 (-0.94 to 1.74) |  |
| Current TDF use | No TDF | 0.28 (-0.70 to 1.26) | 0.3532 |
|  | TDF-containing | -0.33 (-1.48 to 0.81) |  |
| BMI category | Overall effect | — | 0.8870 |
| Calendar period | Overall effect | — | 0.8600 |
|  | 2007-2009 | 0.26 (-1.37 to 1.90) |  |
|  | 2010-2012 | 1.04 (-1.38 to 3.46) |  |
|  | 2013-2015 | -0.11 (-1.38 to 1.15) |  |
|  | 2016-2018 | -0.60 (-2.01 to 0.80) |  |
|  | 2019-2021 | -0.40 (-1.63 to 0.82) |  |
|  | 2022-2024 | -0.35 (-1.73 to 1.03) |  |

*For T1-to-T2, no significant calendar-period effect was observed in the adjusted model. Abbreviations: BMI, body mass index; IDU, injection drug user; CDC, center for disease control and prevention; IQR, interquartile range; ART, antiretroviral therapy; RAL, raltegravir; DRV/r, darunavir/ritonavir; EVG, elvitegravir; RPV, rilpivirine; DTG, dolutegravir; DRV/c, darunavir/cobicistat; DOR, doravirine; BIC, bictegravir; CAB, cabotegravir; TDF, tenofovir disoproxil fumarate; eGFR, estimated Glomerular Filtration Rate*
